# A rise in mean platelet volume during hospitalization for community-acquired pneumonia predicts poor prognosis: a retrospective observational cohort study

**DOI:** 10.1186/s12890-017-0483-6

**Published:** 2017-10-30

**Authors:** Oleg Gorelik, Irma Tzur, Dana Barchel, Dorit Almoznino-Sarafian, Muhareb Swarka, Ilia Beberashvili, Leonid Feldman, Natan Cohen, Shimon Izhakian

**Affiliations:** 1 Department of Internal Medicine “F”, Assaf Harofeh Medical Center, 70300 Zerifin, Israel; 2 Nephrology Division, Assaf Harofeh Medical Center, Zerifin, Israel; 3 Sackler Faculty of Medicine, Tel Aviv University, Ramat Aviv, Tel Aviv, Israel

**Keywords:** Mean platelet volume, Community-acquired pneumonia, Hospitalization, Prognosis

## Abstract

**Background:**

Clinical characteristics and the prognostic significance of changes in mean platelet volume (MPV) during hospitalization for community-acquired pneumonia (CAP) have not been investigated.

**Methods:**

Among 976 adults hospitalized for CAP, clinical characteristics, in-hospital outcomes (transfer to the intensive care unit, treatment with mechanical ventilation, prolonged hospital stay and death), and all-cause mortality following discharge, were compared according to ΔMPV (MPV on discharge minus MPV on admission): groups A (no rising MPV, ΔMPV < 0.6 fL) and B (rising MPV, ΔMPV ≥ 0.6 fL).

**Results:**

Groups A and B comprised 83.8% and 16.2% of patients, respectively. Patients with a rise in MPV were more likely to be older, and to present with renal dysfunction, cerebrovascular disorder and severe pneumonia than were patients with no rise in MPV. On discharge, lower values of platelets and higher levels of neutrophils were observed in group B. Rising MPV strongly predicted a need for mechanical ventilation and in-hospital death (the respective relative risks: 2.62 and 6.79; 95% confidence intervals: 1.54–4.45 and 3.48–13.20). The respective 90-day, 3-year and total (median follow-up of 54 months) mortality rates were significantly higher in group B (29.1%, 43.0% and 50.0%) than group A (7.3%, 24.2% and 32.6%), *p* < 0.001 for all comparisons. A rise in MPV was a powerful predictor of all-cause mortality (relative risk 1.26 and 95% confidence interval 1.11–1.43).

**Conclusions:**

Rising MPV during hospitalization for CAP is associated with a more severe clinical profile than no rise in MPV. A rise in MPV strongly predicts in-hospital and long-term mortality.

## Background

Platelets play an important role in processes of hemostasis, inflammation and immunity [1, 2]. Mean platelet volume (MPV) is a routine laboratory test that is measured in complete blood count and considered a marker of platelet function and activation [3, 4]. A single measurement of elevated MPV has been reported to be associated with increased morbidity and mortality in various patient populations [3–7]. Moreover, MPV is a dynamic parameter that may change significantly within several days or weeks [8–15]. In a number of studies consisting of patients with critical illness [8, 11, 12, 14], bacteremia [10], coronary artery disease [9, 15] and heterogeneous disorders [13], a rise in MPV over time was identified as a powerful predictor of morbidity and mortality.

Patients hospitalized with community-acquired pneumonia (CAP) are at an increased risk of death in the hospital and following discharge [16–18]. The prognostic significance of MPV has been reported in only two small studies on CAP patients, which were based on single MPV determinations [19, 20]. The clinical characteristics and prognosis of time-dependent MPV changes have not been investigated in the CAP population. Therefore, we aimed to compare demographic, clinical, laboratory and radiographic characteristics, as well as short- and long-term outcomes of patients hospitalized for CAP, according to changes in MPV.

## Methods

### Study population and design

The study population comprised adult patients hospitalized for CAP during 2009–2012 in 7 internal medicine departments in our tertiary care university hospital. The availability of complete blood count at admission and within 48 h of discharge or death, and with intervals between the determinations of at least 3 days, was a study inclusion criterion. Patients transferred from another hospital or from an intensive care unit (ICU), with possible health care-associated and nosocomial pneumonia, primary hematological disorders, advanced malignant disease or platelet transfusion were excluded from the analysis. MPV and other blood count parameters were measured by an automated analyzer (Coulter® A84148-AB; Beckman Coulter, Inc., CA, USA) with LH 750 control system. All blood samples were collected, handled and processed in the same way: venous blood was drawn into a test tube containing an anticoagulant (EDTA) and the samples were tested within 60 min of collection. In our laboratory, the range of normal MPV values is 7.3–11.5 fL, and the respective intra- and inter-assay coefficients of variability for MPV measurements are ≤ 2.2% and ≤ 6.3%.

For analysis of time-dependent MPV changes, patients were categorized according to ΔMPV (MPV on discharge minus MPV on admission) into: groups A (no rising MPV, ΔMPV < 0.6 fL) and B (rising MPV, ΔMPV ≥ 0.6 fL). Data were also compared between groups classified according to values of MPV ≤ 8.5 fL and > 8.5 fL on admission (groups 1adm and 2adm, respectively) and discharge (groups 1dis and 2dis, respectively). The study was carried out in accordance with the Declaration of Helsinki and was approved by the institutional Ethics Committee of the Assaf Harofeh Medical Center (approval number 0195–16-ASF).

### Data collection

Demographic, clinical, radiographic and laboratory data were obtained retrospectively from the electronic medical records of the patients. We recorded the following outcomes during the current hospitalization: transfer to ICUs, treatment with mechanical ventilation, length of hospital stay and death. Death of any cause was registered at 90 days and 3 years following the current admission, and at the end of the follow-up period. Information about death was obtained from the registry of the Internal Affairs Ministry or from hospital records.

### Definitions

A ΔMPV ≥ 0.6 fL was chosen to minimize the chance of misclassification of patients due to known normal individual variability or measured counter variability of MPV [4], and according to findings in other relevant investigations [14]. The rationale to use the cut-off of 8.5 fL for MPV values on admission and discharge was based on a previous report in which this threshold was optimal for predicting mortality in CAP patients [20]. Anemia was defined using the World Health Organization criteria: a hemoglobin concentration of < 13 g/dl in men and < 12 g/dl in women. Renal dysfunction was diagnosed according to any value of estimated glomerular filtration rate < 60 ml/min/1.73 m^2^ during current hospitalization, using the Modification of Diet in Renal Disease equation [21].

CAP was diagnosed according to the following criteria: the presence of acute illness with symptoms and signs of lower respiratory tract infection and a new chest radiographic infiltrate; no evidence of another clear diagnosis or of acquirement in a hospital or of being associated with health care [16–18]. Patients were stratified into risk classes according to the Pneumonia Severity Index (PSI) and CURB-65 scores calculated at hospital admission [22, 23].

### Statistical analysis

The statistical analysis was performed using the Biomedical Package (BMDP) software program [24]. The results were expressed as means and standard deviations for quantitative data, and as percentages for qualitative data. Statistical comparisons were performed for groups A vs. B, 1adm vs. 2adm, and 1dis vs. 2dis. Categorical variables were compared using Pearson’s chi-square or Fisher’s exact test. Analysis of Variance (ANOVA) was applied for continuous variables. Survival estimates were provided using the Kaplan-Meier method. Mantel-Cox and Breslow tests were applied to evaluate differences between the curves. *P* values ≤ 0.05 were considered statistically significant. Variables that most significantly predicted poor in-hospital outcomes were evaluated by stepwise logistic regression with determination of the area under the curve (AUC) of the receiver operating characteristic plots. Variables that were found to be associated with shortened survival using the Kaplan-Meier method were reevaluated by the Cox proportional-hazards model to identify those most significantly associated with mortality.

## Results

### Characteristics of the patients

#### Entire sample

Data for the 976 patients who were included in the study are presented in Table 1. Groups A (no rising MPV) and B (rising MPV) comprised 83.8% and 16.2% of the patients, respectively. MPV values > 8.5 fL on admission and discharge were found among 48.3% and 43.0% of patients, respectively.

**Table 1:** Characteristics of the patients analyzed in the study

Characteristics	Entire sample (*n* = 976)	Group A No rising MPV ΔMPV < 0.6 fL (*n* = 818)	Group B Rising MPV ΔMPV ≥ 0.6 fL (*n* = 158)	Difference between groups A and B *p*-value
Age (years)	64.6 ± 21	63.4 ± 20	70.7 ± 21	**< 0.001**
Male sex	52.3%	51.2%	57.6%	0.1
Comorbid conditions				
Renal dysfunction	33.6%	31.3%	45.6%	**< 0.001**
Diabetes mellitus	30.9%	31.4%	28.5%	0.5
Coronary artery disease	24.2%	23.5%	27.8%	0.2
Chronic lung disease	28.1%	27.0%	33.5%	0.1
Heart failure	16.8%	15.8%	22.2%	0.06
Cerebrovascular disease	16.9%	15.2%	25.9%	**0.001**
History of malignancy	8.4%	8.2%	9.5%	0.6
Acute bleeding during hospitalization/blood transfusion	3.3%/6.0%	2.9%/5.7%	5.1%/7.6%	0.5/0.4
Pleural effusion/bilateral lung involvement	25.8%/16.0%	25.8%/15.5%	25.9%/18.4%	1.0/0.4
CURB-65 class	1.78 ± 1.0	1.71 ± 1.0	2.15 ± 1.1	**< 0.001**
Pneumonia Severity Index score/risk class	100.1 ± 45/3.44 ± 1.4	98.3 ± 44/3.36 ± 1.4	117.2 ± 47/3.85 ± 1.3	**< 0.001/< 0.001**
Antibiotic treatment				
Beta-lactam	93.0%	93.0%	93.0%	1.0
Macrolide	62.4%	61.5%	67.1%	0.2
Fluoroquinolone	23.4%	23.5%	22.8%	0.9
Other	11.8%	11.7%	12.0%	0.9
Laboratory data				
Hypoalbuminemia on admission (< 34 g/l)	36.2%	34.8%	39.9%	0.2
Hyponatremia on admission (< 135 mmol/l)	37.9%	39.1%	31.0%	0.06
Hemoglobin on admission/discharge (normal 13.0–16.2 g/dl)	12.5 ± 2/12.0 ± 2	12.5 ± 2/12.0 ± 2	12.4 ± 2/11.7 ± 2	0.4**/0.02**
Anemia on admission/discharge	45.4%/61.4%	44.6%/60.5%	49.4%/65.8%	0.3/0.2
WBC count on admission/discharge (normal 4−11 × 10^9^/l)	12.5 ± 6/9.1 ± 4	12.6 ± 7/8.9 ± 4	12.2 ± 6/10.4 ± 6	0.4/**< 0.001**
Neutrophil count on admission/discharge (normal 2.0–7.7 × 10^9^/l)	10.4 ± 6/6.6 ± 4	10.5 ± 6/6.3 ± 3	10.1 ± 6/8.0 ± 6	0.5**/< 0.001**
Platelet count on admission/discharge (normal 140−450 × 10^9^/l)	229 ± 93/287 ± 122	230 ± 93/300 ± 123	226 ± 93/221 ± 86	0.6/**< 0.001**
MPV on admission/discharge (normal 7.3–11.5 fL)	8.64 ± 1.2/8.48 ± 1.2	8.67 ± 1.3/8.29 ± 1.1	8.44 ± 0.9/9.46 ± 1.1	**0.03**/**< 0.001**
MPV on admission ≤ 8.5/> 8.5 fL	51.7%/48.3%	50.7%/49.3%	57%/43%	0.1
MPV on discharge ≤ 8.5/> 8.5 fL	57%/43%	64.3%/35.7%	19%/81%	**< 0.001**
Duration between MPV measurements (days)	7.4 ± 9	7.2 ± 8	8.3 ± 10	0.2
In-hospital outcomes				
Transfer to intensive care unit	3.8%	3.7%	4.4%	0.6
Mechanical ventilation	9.2%	6.8%	21.5%	**< 0.001**
Duration of hospital stay (days)	8.1 ± 9	7.9 ± 8	9.0 ± 10	0.1
Death	6.3%	3.3%	21.5%	**< 0.001**

Data are presented as means ± SD or percentages of presented cases. *MPV* mean platelet volume; ΔMPV: MPV on discharge minus MPV on admission; CURB-65: assigning 1 point each for confusion, blood urea nitrogen > 19 g/dl, respiratory rate ≥ 30 breaths/min, low blood pressure and age ≥ 65 years; *WBC* white blood cell. Bold entries in the table indicate a *p*-value of ≤ 0.05

#### Comparisons according to MPV changes (group A vs. B, Table 1)

Patients with a rise in MPV were more likely to be older, and to present with renal dysfunction and cerebrovascular disorder than patients with no rise in MPV. Mean values of CURB-65 and PSI scores were significantly higher in group B (rising MPV). Moreover, on discharge, lower values of platelets, as well as higher levels of leukocytes and neutrophils were observed in group B.

### In-hospital outcomes

#### Comparisons according to MPV changes (group A vs. B)

Patients with a rise in MPV were more likely to be treated with mechanical ventilation and to die during the current hospitalization than were patients with no rise in MPV (Table 1). On multivariate analysis, a rise in MPV was one of the variables that strongly predicted treatment with mechanical ventilation and in-hospital death (Table 2).

**Table 2:** Variables that were most significantly associated with in-hospital outcomes in the entire study population (stepwise logistic regression analysis)

In-hospital outcomes	*p*-value	Relative risk	95% confidence interval
Transfer to intensive care unit (AUC = 0.73)			
Age^a^	0.011	0.587	0.46–0.75
Pneumonia Severity Index score^b^	< 0.001	2.75	1.63–4.65
Coronary artery disease	0.027	3.00	1.32–6.82
Cerebrovascular disease	0.011	0.169	0.38–0.76
Hypoalbuminemia on admission	0.020	2.56	1.26–5.21
Mechanical ventilation (AUC = 0.86*)*			
Pneumonia Severity Index score^b^	< 0.001	4.27	2.98–6.11
ΔMPV ≥ 0.6 fL	< 0.001	2.62	1.54–4.45
Chronic lung disease	0.04	1.76	1.07–2.90
Cerebrovascular disease	0.037	1.70	1.00–2.56
Prolonged (> 7 days) hospital stay (AUC = 0.72)			
Pneumonia Severity Index score^b^	< 0.001	2.28	1.89–2.77
Chronic lung disease	0.004	1.61	1.18–2.21
Hypoalbuminemia on admission	0.01	1.44	1.06–1.95
Female sex	0.015	1.37	1.02–1.85
In-hospital death (AUC = 0.93)			
ΔMPV ≥ 0.6 fL	< 0.001	6.79	3.48–13.20
Cerebrovascular disease	< 0.001	3.96	2.03–7.73
Pneumonia Severity Index score^b^	< 0.001	3.90	2.29–6.64
Age^a^	0.004	1.73	1.17–2.55

AUC: the area under the curve of the receiver operating characteristic plots; ΔMPV: MPV (mean platelet volume) on discharge minus MPV on admission. ^a^For each 10 year increment. ^b^For each 50 points increment

#### Comparisons according to MPV at admission (group 1adm vs. 2adm) and discharge (group 1dis vs. 2dis)

Patients in group 2adm were more often mechanically ventilated than were those in group 1adm (12.1% vs. 6.5%, *p* = 0.003). Treatment with mechanical ventilation (13.8% vs. 5.8%, *p* < 0.001) and in-hospital death (10.0% vs. 3.4%, *p* < 0.001) were more likely observed in patients from group 2dis than in those from group 1dis. No statistically significant differences in any other in-hospital outcomes were found between the groups (data are not presented). On multivariate analysis, none of the in-hospital outcomes were associated with MPV on admission or discharge.

### Survival

#### Entire sample

The follow-up period extended up to 85 months (median 54 months). The respective 90-day, 3-year and total mortality rates for the entire sample were 11.2%, 27.6% and 35.5%.

#### Survival according to MPV changes (group A vs. B)

**Fig. 1:**
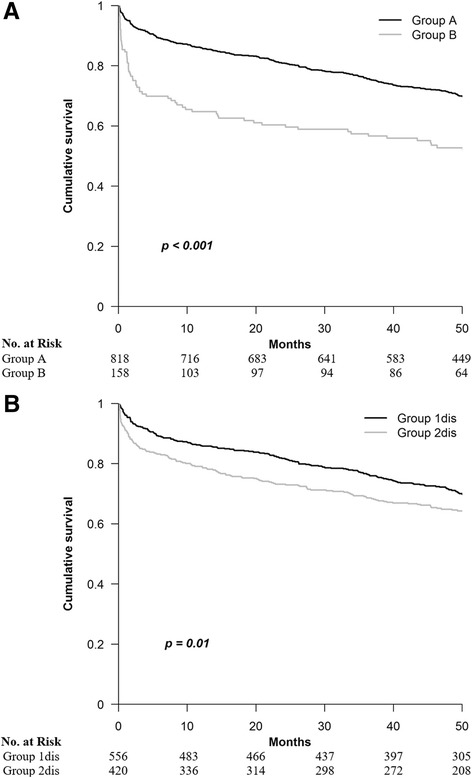
The Kaplan-Meier estimates of survival for the various study groups. **a** − Association between ΔMPV and survival. Group A − no rising MPV (ΔMPV < 0.6 fL). Group B − rising MPV (ΔMPV ≥ 0.6 fL). **b** − Association between MPV on discharge and survival. Group 1 dis − MPV ≤ 8.5 fL. Group 2 dis − MPV > 8.5 fL on discharge. MPV: mean platelet volume; ΔMPV: MPV on discharge minus MPV on admission

Rising compared to not rising MPV was associated with increased all-cause mortality (*p* < 0.001, Fig. 1a). The respective 90-day, 3-year and total mortality rates were significantly higher in group B (29.1%, 43.0% and 50.0%) than in group A (7.3%, 24.2% and 32.6%), *p* < 0.001 for all comparisons.

#### Survival according to MPV at admission (group 1adm vs. 2adm)

No significant difference in survival was found between groups 1adm and 2adm: the respective 90-day, 3-year and total mortality rates were 11.5%, 27.3% and 36.8% vs. 10.2%, 27.2% and 34.0% (*p* > 0.5 for all comparisons).

#### Survival according to MPV at discharge (group 1dis vs. 2dis)

Mortality was higher among patients from group 2dis than among those from group 1dis (*p* = 0.01, Fig. 1b): the respective rates were 15.0% vs. 7.7% at 90 days (*p* < 0.001), 31.4% vs. 24.1% at 3 years (*p* = 0.01) and 39.0% vs. 32.7% at the end of follow-up (*p* = 0.01).

#### The variables most significantly associated with decreased survival in the entire sample

Univariate analysis revealed that advanced age, male sex, rise in MPV, MPV > 8.5 fL at discharge, anemia, renal dysfunction, diabetes mellitus, coronary artery disease, chronic lung disease, heart failure, cerebrovascular disease, history of malignancy, hyponatremia, hypoalbuminemia, and higher CURB-65 and PSI scores were associated with increased long-term mortality. Reevaluation of these variables by the Cox proportional-hazards model, separately for PSI and CURB-65 scores, showed that a rise in MPV was a powerful predictor of shortened survival in both models (the respective relative risks: 1.26 and 1.23, Table 3). However, elevated MPV at discharge did not remain significantly associated with mortality.

**Table 3:** Variables that were most significantly associated with low survival in the entire cohort (Cox proportional-hazards model)

Variable	*p*-value	Relative risk	95% confidence interval
Model with Pneumonia Severity Index score			
Age^a^	< 0.001	1.60	1.44–1.79
Pneumonia Severity Index score^b^	< 0.001	1.85	1.53–2.22
Cerebrovascular disease	< 0.001	2.15	1.68–2.74
ΔMPV ≥ 0.6 fL	0.001	1.26	1.11–1.43
History of malignancy	0.001	1.78	1.31–2.44
Heart failure	0.003	1.46	1.13–1.88
Hypoalbuminemia on admission	0.009	1.43	1.14–1.78
Chronic lung disease	0.015	1.32	1.06–1.64
Model with CURB-65 class			
Age^a^	< 0.001	1.75	1.58–1.95
Cerebrovascular disease	< 0.001	2.37	1.86–3.02
Heart failure	< 0.001	1.77	1.39–2.25
CURB-65^c^	< 0.001	1.30	1.14–1.49
History of malignancy	< 0.001	1.86	1.36–2.55
ΔMPV ≥ 0.6 fL	0.001	1.23	1.08–1.41
Hypoalbuminemia on admission	0.003	1.44	1.16–1.80
Chronic lung disease	0.006	1.36	1.09–1.69
Female sex	0.019	0.77	0.62–0.96

ΔMPV: MPV (mean platelet volume) on discharge minus MPV on admission; CURB-65: assigning 1 point each for confusion, blood urea nitrogen > 19 g/dl, respiratory rate ≥ 30 breaths/min, low blood pressure and age ≥ 65 years. ^a^For each 10 year increment. ^b^For each 50 points increment. ^c^For each 1 point increment

## Discussion

The present investigation is the first report of clinical characteristics and prognosis associated with MPV changes during hospitalization for CAP. The main novelty of our study is the demonstration that a rise in MPV strongly predicts in-hospital and long-term mortality.

Information about prognostic significance of MPV among CAP patients is limited [19, 20]. In one study comprising 196 children with CAP, baseline MPV values were significantly higher in hospitalized patients than in outpatients [19]. In another investigation of 174 adults presenting with CAP to an emergency department, MPV values below a cut-off of 8.55 fL, in combination with a CURB-65 score, strongly predicted 28-day all-cause mortality [20]. Compared to the aforementioned studies, the current investigation included a larger sample size and hospitalized patients only. Moreover, in addition to admission, we determined MPV values at discharge and MPV changes during hospitalization. Finally, we evaluated in-hospital and long-term outcomes. Using a similar cut-off for MPV values as in the study of Golcuk et al. [20], we did not find a significant difference in in-hospital and long-term mortality rates between patients with baseline MPV levels below and above the threshold. Interestingly, MPV values above the cut-off at discharge were associated with an increased risk of mechanical ventilation and death during the current hospitalization, and shortened survival following discharge. However, in multivariate analysis, MPV at discharge was not one of the variables most significantly associated with poor outcomes. Thus, in our patient population, MPV determined once at any point of time probably appears as a marker of the severity of pneumonia and comorbidities, rather than as an independent predictor of prognosis.

A strength of this study is the evaluation in detail of demographic, clinical, laboratory and radiographic characteristics associated with MPV changes during hospitalization for CAP. Information regarding the association of clinical characteristics with dynamic MPV changes is scarce. In three available studies of patients with coronary artery disease and sepsis, rising MPV was associated with increasing age, renal failure and peripheral artery disease [9, 14, 15]. We also observed that rising MPV is associated with age and renal dysfunction. Moreover, our patients with a rise in MPV were more likely to present with cerebrovascular disorder and severe pneumonia than patients with no rise in MPV. The main potential mechanism for rising MPV in our patient population is severe inflammation caused by pneumonia. In severe infection, increased release of thrombopoietin and various inflammatory cytokines, such as interleukin-1, −3 and −6 and tumor necrosis factor-α, result in increased thrombopoiesis and enhanced expression of younger large platelets into the blood circulation [2, 4, 8, 10]. On the other hand, rising MPV may be attributed to increased thrombocyte consumption in the peripheral tissue and spleen, induced by severe inflammatory status [4, 8, 10]. Indeed, in our patient population, a rise in MPV was associated with higher pneumonia severity scores, higher levels of leukocytes and neutrophils at discharge, and lower platelet counts on discharge. Another possible contributing factor to a rise in MPV in our patients is renal dysfunction which is known to be associated with increasing MPV [14, 25]. Hypoxemia, which may cause increased platelet consumption and bone marrow stimulation is an additional explanation for a rise in MPV [11].

The prognostic significance of time-dependent MPV changes has not been reported in patients with pneumonia. Our most interesting finding is the demonstration that a rise in MPV during hospitalization for CAP, even to values within the normal range, predicted treatment with mechanical ventilation and hospital death, as well as increased mortality following discharge from the hospital. Moreover, rising MPV was the most powerful predictor for in-hospital death. A rise in MPV less strongly predicted increased long-term mortality than did advanced age, cerebrovascular disease and higher PSI and CURB-65 scores, but it remained one of variables that was most significantly associated with shortened survival. In contrast to other powerful predictors of poor prognosis that were determined on hospital admission, the ΔMPV reflected dynamic MPV changes throughout hospitalization. Thus, we suggest that repeated assessment of a simple routine parameter such as MPV during hospitalization may provide additional prognostic information and improve risk stratification for CAP patients. Our findings are in concordance with the results of a number of studies in various patient populations and with various designs [8–15]. Firstly, a rise in MPV in the first 24 h after admission to the ICU [12] and during hospitalization in the ICU [8, 11] was associated with higher hospital mortality. Secondly, MPV elevation during hospitalization for sepsis [14] and within 3 weeks after bacteremia [10] strongly predicted 30-day mortality. Finally, rising MPV within 24 h after admission for acute myocardial infarction [15], during hospitalization in an internal medicine ward [13] and 3 years following percutaneous coronary intervention [9] was associated with increased long-term mortality.

The underlying pathophysiological mechanisms for a relationship of rising MPV with poor prognosis are not fully understood. The main potential explanation is increased platelet activation [1–4]. Larger thrombocytes are known to be functionally, metabolically and enzymatically more active than smaller ones [1–4]. The greater activation of enlarged platelets results in increased release of procoagulant substances such as thromboxane A2, β-thromboglobulin and surface proteins [1–4]. Consequent hyperaggregability of platelets, extended vasoconstriction and endothelial dysfunction may contribute to an increased short-term risk of cardiovascular thrombosis and death in patients with rising MPV [3, 4]. Moreover, large thrombocytes more actively secrete from their granules cytokines, enzymes, oxidants and other proteins, which may decrease immune function of platelets and other circulating cells, and increase detrimental processes of oxidation and apoptosis [1, 2, 11]. We suggest that the abovementioned negative effects of persistent increased activation of enlarged platelets following acute infection is a possible underlying mechanism of increased long-term mortality in our patients.

The present investigation has a few limitations. First, this is a single-center study. Furthermore, the retrospective design precludes determining a definitive relationship between MPV changes and outcomes. Finally, possible selection bias and missing data, which is typical of observational research, may have impacted the results.

## Conclusions

Rising MPV during hospitalization for CAP is associated with more severe clinical and laboratory characteristics than no rise in MPV. Rising MPV is a powerful predictor of in-hospital and long-term mortality. We suggest that repeated MPV determinations throughout hospitalization may improve risk stratification for CAP patients.

## Abbreviations

ANOVA: Analysis of variance
AUC: Area under curve
BMDP: Biomedical package
CAP: Community-acquired pneumonia
CURB-65: assigning 1 point each for Confusion, blood Urea nitrogen >19 g/dl, Respiratory rate ≥ 30 breaths/min, low Blood pressure and age ≥ 65 years
ICU: Intensive care unit
MPV: Mean platelet volume
PSI: Pneumonia severity index
WBC: White blood cell
